# Supplementary material for: Crumpling Damaged Graphene
Source: Sci Rep. 2016 May 13;6:25891. doi: 10.1038/srep25891 (PMC4865728; doi:10.1038/srep25891)
Supplement: Supplementary Information [file srep25891-s1.pdf]

Supplementary Material: Crumpling Damaged Graphene

I.Giordanelli[1,*], M. Mendoza[1], J. S. Andrade, Jr.[1,2], M. A. F. Gomes[3], and H. J. Herrmann[1,2]

[1]ETH Zürich, Computational Physics for Engineering Materials, Institute for Building Materials, Wolfgang-Pauli-Strasse 27, HIT, CH-8093 Zürich, Switzerland
[2]Universidade Federal do Ceará, Departamento de Física, Campus do Pici, 60455-760 Fortaleza, Ceará, Brazil
[3]Universidade Federal de Pernambuco, Departamento de Física, 50670-901 Recife-PE, Brazil
[*]gilario@ethz.ch

ABSTRACT

This supplementary material contains some additional details regarding the fractal dimension measure, the simulation set-up, the anisotropy and asphericity of crumpled damaged graphene sheets, the contribution of newly formed covalent bonds and the derivation of the theoretical model presented in the paper.

Fractal dimension

In order to determine the fractal dimension systematically and consistently, in the case of the sand-box method, we have to determine first the power-law range for each DGM separately. A least-squares fit to the data is then applied and the resulting exponents are averaged over all DGM realizations.

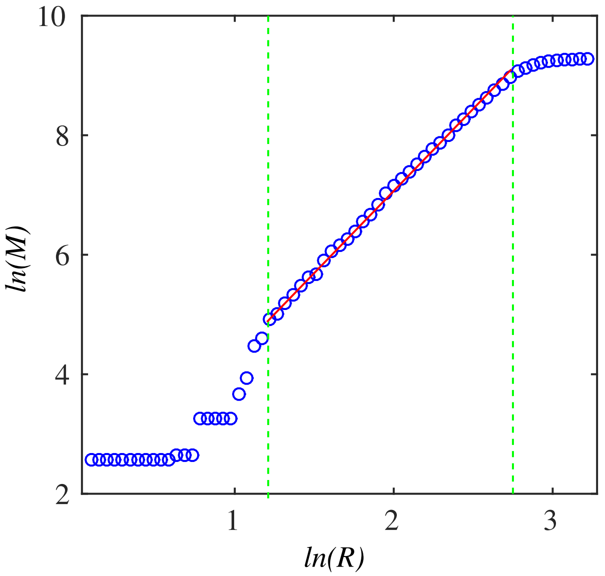

Figure S1. Typical example of the fitting used to obtain the fractal dimension with the sand-box method. Here, we adopt aR_g, with $a = 0.19$, and R_g as appropriate values for the lower and upper threshold, respectively. The dashed green lines delineate the thresholds of the scaling region. The fractal dimension for this particular case is 2.71 ± 0.03 and corresponds to a graphene sheet with $p = 0.28$.

In Fig. S2 we show the largest sphere used by the sand-box method. The structure is between the flat and the crumpled state. We clearly see that the DGM is still flat with some ripples and the spheres of the sand-box method contain regions where there are no atoms. On the contrary, in Fig. S3 we see that the sphere is completely contained in the DGM.

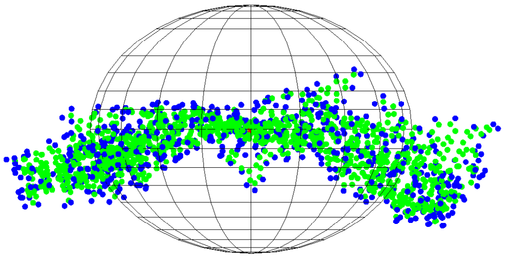

Figure S2. DGM at $p = 0.16$. The green points represent carbon atoms, the blue points hydrogen atoms and the red point the center of mass. The radius of the sphere corresponds to the radius of gyration.

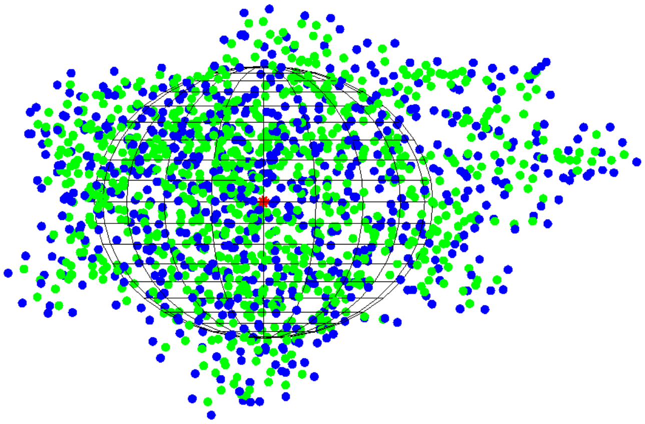

Figure S3. DGM at $p = 0.28$. The green points represent carbon atoms, the blue points hydrogen atoms and the red point the center of mass. The radius of the sphere corresponds to the radius of gyration and covers the isotropic core of the structure.

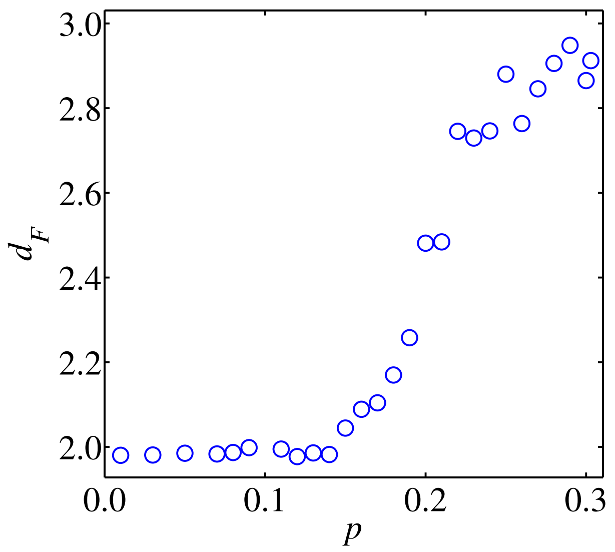

Figure S4. Fractal dimension computed with Eq. (3) (ensemble method) for sheets of different initial sizes ranging from 24 to 60 with NCIs.

Ensemble method

We provide further evidence that the crumpled DGM is fractal using an additional method, which we will call the ensemble method. It is based on the relation between the radius of gyration R_g and the mass M of the DGM as expressed by Eq. (3). Different system sizes are required for this method. Small system sizes have a very small isotropic core and are thus not appropriate for measuring the fractal dimension. In contrast, large system sizes would produce accurate results but at a very high computational cost. Therefore we focus on intermediate system sizes in the range of $24-60$ Å. We observe that the fractal dimension increases in region II as it does with the sand-box method (see Fig. S4). However, the plateau obtained with the sand-box method (seen in region III of Fig. 4) is more pronounced than with the ensemble-method seen in Fig. S4.

Cooling process and fractal dimension

From Fig. S5 we see that as we decrease the temperature of the samples we converge to the fractal dimension d_F.

Computational details

All the parameters used in the simulation have been summarized on Table 1. The initial temperature T_i has been chosen to be 800 K, because it is low enough to keep covalent bonds, avoiding that some atoms would detach from the DGM. However, it is sufficiently high for achieving a fast equilibration process and helps the DGM to explore a broader part of the phase space with configurations that require a high kinetic energy for overcoming some possible local minima configurations. Note also that the initially constructed configuration is not an energetically favourable configuration and a high temperature (and thus high velocity of the atoms) helps to equilibrate faster towards a more natural conformation described by the potential. Subsequently, we cool down the structure gradually using a temperature step size of Δ_T^1 in the temperature interval T_1 and Δ_T^2 in the temperature interval T_2 (see Figure S6). The higher the temperature steps size, the longer it takes for the simulation to achieve the new equilibrium. However, the smaller the temperature step size, the more intervals between 800 K and 0 K we need. We found that $\Delta_T^1 = 5$ K is a good compromise. We defined the second interval T_2 in order to achieve a better precision in the last 25 K. The amount of time-steps required for equilibration strongly depends on the system size. Furthermore, for DGM, the equilibration process is dominated by the carbon atoms, due to their higher mass m_c compared to the hydrogen atoms. The hydrogen atoms play a minor role in the equilibration process. Therefore, we have chosen the equilibration time (Δ_t^1, Δ_t^2) in each temperature interval to be proportional to the number of carbon atoms (N_c) contained in the DGM. The pre-factors have been determined empirically based on the energy evolution. Once we achieve an equilibrium, the energy end temperature fluctuates around the desired value and the velocity of each atom is determined by a Maxwell-Boltzmann distribution. Note that also the free parameter Q in the Nosé-Hoover thermostat determines the coupling between the heat reservoir and the DGM, thus influencing also the temperature fluctuations. This parameter is usually found empirically, based on the fact that a too high value of Q leads to a poor temperature control and for $Q \to \infty$ it even leads to the micro-canonical ensemble (NVE) without

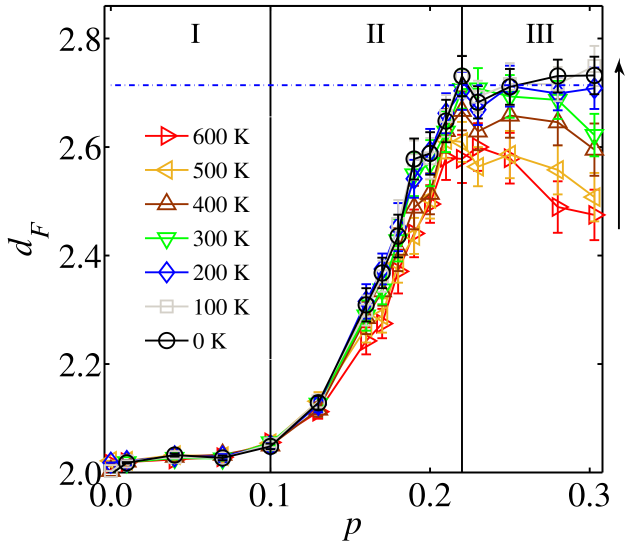

Figure S5. Convergence of the fractal dimension during the cooling process computed with the sand-box method for different vacancy densities p. The arrow is in direction of the cooling process towards 0 K. The dashed blue line indicates the fractal dimension after thermalisation, i.e. $d_F = 2.71 \pm 0.02$.

any temperature control. On the other hand, too small values might induce high-frequency temperature oscillations. In our study we want to produce a structure based on hydrocarbon molecules, where the underlying lattice is hexagonal. Therefore, a natural choice of the initial bond-distance b^i_{cc} would be close to a stable known configuration, in this case a structure close to the one of graphene. During the simulation, the bond-lengths adjust dynamically in order to minimize the total energy and are not fixed parameters of the simulations. Therefore, the precise value of the initial bond-length is not relevant, as long as it is in a suitable range where we have stable covalent bonds between the atoms. In order to be on the safe side, we choose them to coincide with the minimum energy distance predicted for a 100 Å x 100 Å graphene sheet, which is 1.40 ± 0.02 Å as shown in the Figure S7. Indeed, it is matching with the values obtained from many numerical and experimental results[?] (the average bond-length in experimentally grown graphene is found to be 1.42 Å). Note that this argument can be applied to the carbon-hydrogen bonds. We choose an initial bond-length of 1 Å, which is not far away from the equilibrium bond-length between carbon and hydrogen atoms. Note that we could have also started with a bond-length of 1.38 Å or 1.42 Å and this would not have made any difference after reaching thermal equilibrium (We relaxed a graphene sheet with initial bond-length of 1.35 Å and achieved the same equilibrium bond-length mentioned above). However, if we would start with a completely different bond-length for a honeycomb lattice, let's say 2 Å, then the initial configuration will recombine towards a completely different structure or even to amorphous carbon. This process will only be initially dominated by van der Waals forces, since this distance is too large for the covalent bond interaction in carbon. Even though this set-up might lead to interesting results, it is not the scope of this research.

Anisotropy and asphericity

The eigenvalues of the gyration tensor are also useful for computing several shape descriptors. Shape descriptors of particular interest are the relative shape anisotropy κ^2 and asphericity b (see Fig. S8) . The former is a measure for anisotropy and can reach values between 0 and 1. The value 0 occurs when all particles are spherically symmetric and thus the structure is completely isotropic, 0.25 occurs when the particles lie on a plane, and 1 occurs when all points lie on a line. We observe that κ^2 reaches a value of 0.25 for $p = 0$ and decreases for higher values of p, providing evidence that the DGM is more isotropic for large values of p. The asphericity b, which is the second shape descriptor considered, is a quantity that is 0 for particles that are spherically symmetric. We observe that the asphericity is also decreasing as a function of p with a turning point in region II, which is the region where the DGM crumples.

Contribution from new covalent bonds

In this section we study the evolution of the covalent bonds. It is reasonable to believe that possibly newly-formed covalent bonds increase the compactness of the structure. It might be also possible that during the evolution, some new covalent bonds

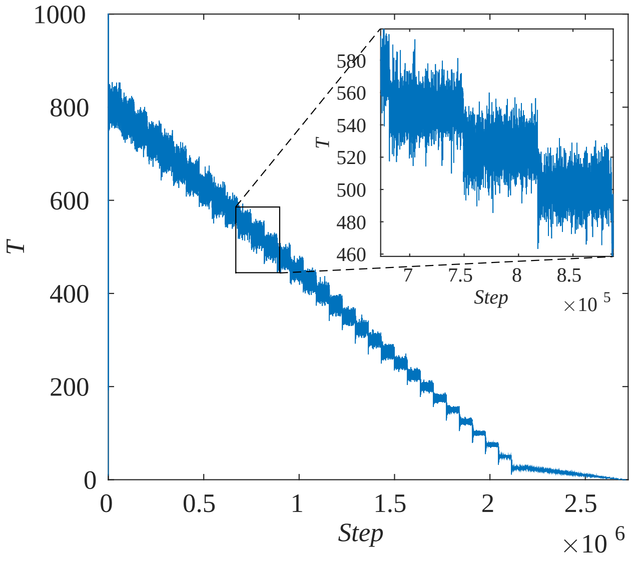

Figure S6. Temperature evolution of a DGM with $p = 0.13$ Å. The DGM has 1193 carbon atoms and 543 hydrogen atoms. We clearly see that after each change of temperature we leave enough time to equilibrate again.

Parameter	Description
$m_c = 12$ u	Mass in atomic units for carbon atoms
$m_h = 1$ u	Mass in atomic units for hydrogen atoms
$b^i_{cc} = 1.4$ Å	Initial bond-length of carbon-carbon bonds
$b^i_{ch} = 1.0$ Å	Initial bond-length of carbon-hydrogen bonds
$\sigma_{cc} = 3.4$ Å	Lennard Jones parameter for carbon carbon interaction
$C_t = 2.5\sigma_{cc}$	Cutoff length for the potential
N_c	Carbon atoms
$\Delta t = 0.1$ fs	Time step
$T_i = 800$ K	Initial temperature
$800\text{ K} \geq T_1 \geq 25\text{K}$	Temperature interval 1
$25\text{ K} \geq T_2 \geq 0\text{K}$	Temperature interval 2
$\Delta^1_T = 25$ K	Temperature step difference in T_1
$\Delta^2_T = 5$ K	Temperature step difference in T_2
$\Delta^1_t = 57N_c$	Time steps for each interval T_1
$\Delta^2_t = 17N_c$	Time steps for each interval in T_2
$Q = N_c/65000$	Free parameter for the Nosé-Hoover thermostat

Table 1. Complete table with the parameters required to reproduce the molecular dynamics simulation.

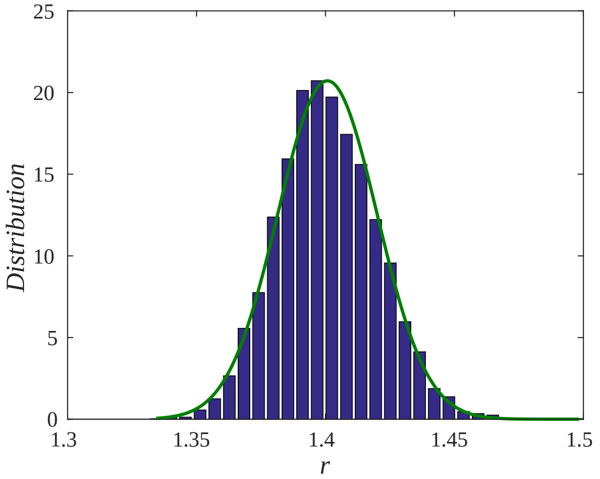

Figure S7. Distribution of the covalent bonds for a relaxed graphene sheet of 100 x 100 Å size. The green line denotes the fit with mean 1.40 Å and standard deviation of 0.02 Å.

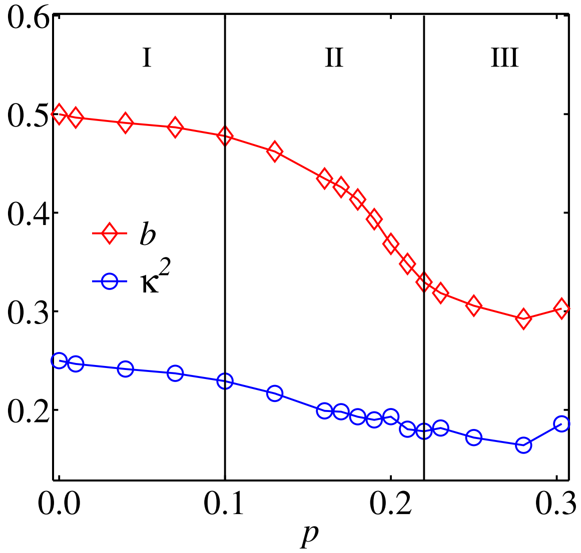

Figure S8. Relative shape anisotropy κ^2 and asphericity b computed with the normalized eigenvalues λ_n/R_g^2.

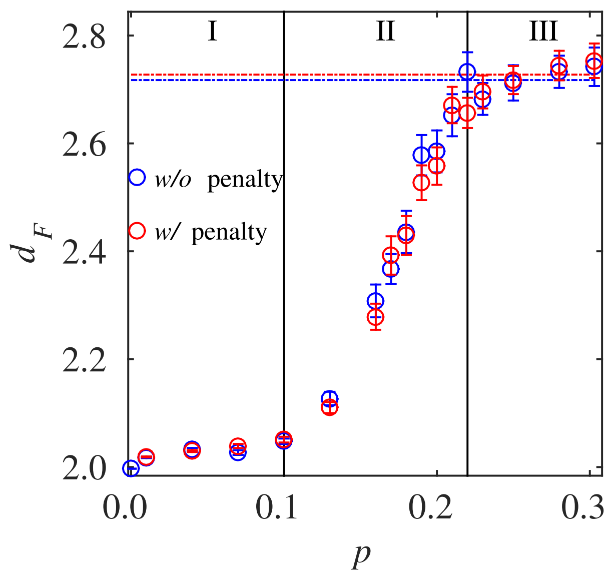

Figure S9. Fractal dimension of the modified DGM, where a penalty has been introduced to suppress newly formed covalent bonds.

are temporarily created. They could play an important role in the crumpling transition.

To address these questions we modified the simulation such that, whenever new covalent bonds are formed, a force is produced that, tries to separate the covalent bonds again. This does not allow the DGM to keep newly-formed covalent bonds. Figs. S9 and S10 clearly show that the impact of newly formed covalent bonds is negligible. There are some new bonds which are created, but they disappear again some time steps later.

Theoretical model

An effective theory can be formulated to explain the fractal dimensions obtained from our simulation results. The mass-size scaling $M \propto R^{2.3}$ valid for DGM in absence of NCIs is reminiscent of many other situations including DLA growth,[?] aggregating proteins,[?] crumpled paper,[?] ensemble average of large groups of proteins,[?] and DNA in chromosomes,[?,?] all of them satisfying $M \propto R^{2.5}$, differing $\approx 8\%$ in the scaling exponent. The recurrence of d_F for these structures, in spite of chemical and physical peculiarities, suggests a common underlying origin. In fact, a mean-field phenomenological model using an entropic elastic energy $U_S = AR^2$,[?] and a two-body repulsive energy $U_{SA} = B\rho^2 V$ can explain this scaling. In the last term, ρ is the average density given by mass/volume$= M/V$, with $V \propto R^3$, and consequently, $U_{SA} = BM^2/R^3$. The entropic energy favours collapsed configurations of the system, but the second term, due to self-avoidance interactions, favours open, more extended, configurations of the sheet. Physically, the prefactor A in U_S is expected to depend on the percentage of vacancies as p^z, with $z > 0$; that is, the entropy of the sheet increases with the damage level. After minimisation of $E = U_S + U_{SA}$ with respect to R, the mass-size relation $M \propto R^{2.5}$ follows straightforwardly. If NCIs expressed through a modified Lennard-Jones potential are present, their attractive part that decays as $1/R^6$ tends to combine with the positive U_{SA} term leading to a repulsive potential that effectively behaves more singularly. Our candidate for this composition of energies is a four-body energy which decays as $U_{SA}^{NCI} = C\rho^4 V = CM^4/R^9$. According to this qualitative model, in order to explain the crumpling transition observed in DGM in the presence of NCI, we conjecture that the relevant energy E of the damaged sheet has the form $E = AR^2 + CM^4/R^9$, which after minimisation leads to $M \propto R^{2.75}$, in conformity with Fig. 4, i.e. the attractive part of the non-covalent interaction favours more compact conformations with a greater fractal dimension, and the radius of gyration $R_g = (9C/2A)^{1/11} M^{4/11}$ is expected to scale inversely with p (as $p^{-z/11}$), as suggested by Fig. 2. For completeness, Fig. 4 shows that there is reversibility between the processes leading to the conformations with fractal dimensions $d_F = 2.71 \pm 0.02$ and $d_F = 2.30 \pm 0.05$, i.e. with and without NCIs, respectively. Thus, if the steady-state configuration is initially obtained with NCI, the sudden elimination of this interaction restores the less compact structure with $d_F = 2.30$, and vice-versa.

References

1. Fasolino, A., Los, J. H. & Katsnelson, M. I. Intrinsic ripples in graphene. *Nat. Mater.* **6**, 858–861 (2007).

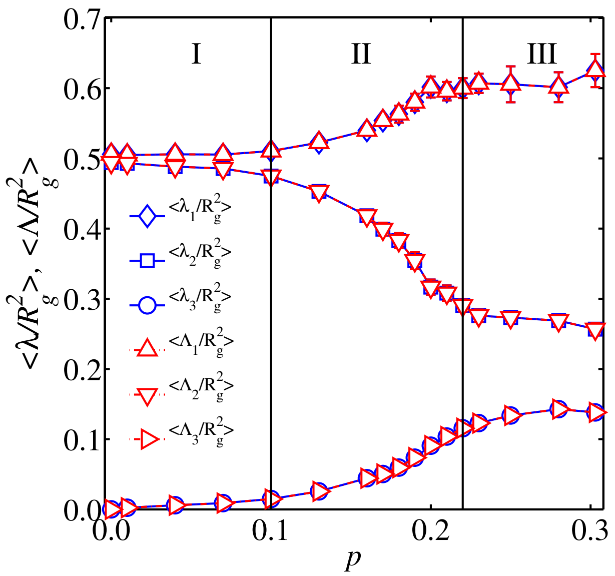

Figure S10. Radius of gyration of the modified DGM, where a penalty has been introduced to suppress newly formed covalent bonds. Blue and red symbols refer to simulations with all and without non-covalent interactions, respectively.

2. Meakin, P. Diffusion-controlled cluster formation in 2-6-dimensional space. *Phys. Rev. A* **27**, 1495–1507 (1983).

3. Feder, J., Jøssang, T. & Rosenqvist, E. Scaling behavior and cluster fractal dimension determined by light scattering from aggregating proteins. *Phys. Rev. Lett.* **53**, 1403 (1984).

4. Gomes, M. A. F. Paper crushes fractally. *J. Phys. A: Math. Gen.* **20**, L283 (1987).

5. Enright, M. B. & Leitner, D. M. Mass fractal dimension and the compactness of proteins. *Phys. Rev. E* **71**, 011912 (2005).

6. Bancaud, A., Lavelle, C., Huet, S. & Ellenberg, J. A fractal model for nuclear organization: current evidence and biological implications. *Nucleic Acids Res.* **40**, 8783 (2012).

7. Metze, K. Fractal dimension of chromatin: potential molecular diagnostic applications for cancer prognosis. *Expert Rev. Mol. Diagn.* **13**, 719–735 (2013).

8. Keten, S. & Buehler, M. J. Strength limit of entropic elasticity in beta-sheet protein domains. *Phys. Rev. E* **78**, 061913 (2008).
